# Battlefield ethics training: integrating ethical scenarios in high-intensity military field exercises

**DOI:** 10.3402/ejpt.v5.23668

**Published:** 2014-08-14

**Authors:** Megan M. Thompson, Rakesh Jetly

**Affiliations:** 1 Defense Research and Development Canada, Toronto, Canada; 2 Canadian Forces Health Services, Ottawa, Canada

**Keywords:** military ethics, battlefield ethics training, ethics field exercises

## Abstract

There is growing evidence that modern missions have added stresses and ethical complexities not seen in previous military operations and that there are links between battlefield stressors and ethical lapses. Military ethicists have concluded that the ethical challenges of modern missions are not well addressed by current military ethics educational programs. Integrating the extant research in the area, we propose that scenario-based operational ethics training in high-intensity military field training settings may be an important adjunct to traditional military ethics education and training. We make the case as to why this approach will enhance ethical operational preparation for soldiers, supporting their psychological well-being as well as mission effectiveness.

The profession of arms is fundamentally moral in nature as it implicates foundational values and principles that have significant impact on the well-being of others (De Cremer, 2009; Heekeren et al., 2005; Jones, 1991; Velasuez & Rostankowski, 1985). Perhaps more than any other profession, the moral decisions and behaviors made in a military context can have profound effects on the military decision maker, their subordinates and peers, their adversaries, and civilians caught in a conflict (Nilsson, 2010; Robinson, 2009), as well as on mission effectiveness and support for the military (Stouffer & Seiler, 2010; Warner & Appenzeller, 2011). Indeed, the state-granted powers of ultimate destruction mean that “the military has a unique obligation to be constrained by moral integrity and competence” (Davenport, 1997, p. 3).

Although military operations have always involved moral dimensions, recent missions have additional complexities in this regard. Current and anticipated future missions have been characterized as “not really ‘war’ at all: but rather as unconventional, asymmetric conflicts with shadowy, elusive and ill-defined enemies and morally ambiguous objectives that are more akin to ongoing attempts to combat organized crime, or stop gang warfare, or identify and arrest drug dealers and human traffickers than they are to armies defending their nations against enemy states in conventional war” (Lucas, 2009, p. xv; see also Robinson, 2009). Insurgents rarely wear uniforms, retreat into the safety of local populations, and often adhere to a set of moral values that are not only inconsistent with, but in fact “… often [deliberately] play against the ethical standards that western societies hold dear” (Cossar, 2008, p. 29) in order to provoke disproportionate retaliation from Western forces. There are also cultural differences with the wider local population, creating additional cultural stress (Azari, Dandeker, & Greenberg, 2010) for military members who have increasing contact with local populations (Riedel, 2008; Warner & Appenzeller, 2011). Ethnic cleansing and atrocities among warring factions have become commonplace (Breed, 1998), and restrictive rules of engagement have meant that intervening militaries can do little more than bear witness to the carnage around them (Dallaire, 2002; Everts, 2000; Litz, Orsillo, Friedman, Ehlich, & Batres, 1997; Thompson & Gignac, 2002; Weisaeth, 2003). Finally, militaries are often called upon to assume combat, humanitarian, and stabilization roles all in the same mission. These shifting mission mandates increase role ambiguity (Adler, Litz, & Bartone, 2003), and ethical challenges (Robinson, 2009). Such added complexity and ambiguity mean that military personnel are called upon to make moral decisions under some of the most challenging of conditions: 1) when the right thing to do is not immediately clear; 2) when two or more important principles or values support different actions, and 3) when some harm will result, regardless of the actions taken (Defense Ethics Program, Department of National Defense, 2012).

Of course, these new operational realities occur within the context of traditional military stressors. These include time pressure, incomplete or ambiguous information, sleep deprivation, and primitive living conditions (Orasanu & Backer, 1996; Thompson & McCreary, 2006; Warner & Appenzeller, 2011). Similarly, fear (Horne, 2004) and the “recognition of one's own destructive capacity and concern about failing one's comrades” (Orasanu & Backer, 1996; Banks, 2012) are never far from mind. Together, these factors create “the threatening psychological ambiance of combat” (Novaco, Cook, & Sarason, 1983, p. 381).

The effects of physical and mental stress on decision making are wide-ranging (Hancock & Szalma, 2008), including a reduced ability to engage in effortful thinking, a greater influence of emotions, and greater automatic information processing (Driskell, Salas, & Johnson, 2006; Kahneman, 2003). Other deficits include an increase in attentional lapses and magical thinking (where beliefs conflict with the laws of nature), the narrowing of perceptual focus, short-term memory impairment, and a greater use of bias and heuristics in decision making (Artwohl & Christensen, 1997; Chajut & Algom, 2003; Keinan, 1987, 1994; Keinan, Friedland, & Ben-Porath, 1987; Leach, 2005; Messervey, 2013; Morgan, Doran, Steffian, Hazlett, & Southwick, 2006; Norenzayan & Hansen, 2006; Orasanu & Backer, 1996; Wickens, 1987; Wickens & Flach, 1988). Each of these has the potential to lead to significant errors in judgment and performance.

Although the effect of stressors on moral decision making is less understood, recent research shows that stressors commonplace in military operations may well affect moral decision making. For instance, sleep deprivation has been associated with decreased ability to recognize a moral issue (Barnes, Schaubroeck, Huth, & Ghumman, 2011; Kjellevold Olsen, Pallesen, & Eid, 2010) and longer decision latency—although it did not affect moral decision quality at least in one study (Killgore et al., 2007). Higher cognitive load (Greene, Morelli, Lowenberg, Nystrom, & Cohen, 2008) and reduced self-control (Gino, Schweitzer, Mead, & Ariely, 2011) have also been shown to interfere with certain types of moral judgments and the recognition of moral issues, respectively. Remarkably, even feelings of cleanliness (through the act of washing one's hands or exposure to words associated with cleanliness) have been shown to have an impact on at least certain moral judgments (Schnall, Benton, & Harvey, 2008).

Together, this evidence underscores the importance of exploring the role of stress on military moral decision processes. It also lends credence to the calls of researchers who question an exclusive reliance on traditional models of moral decision making that are based exclusively on rational, effortful cognition (Rogerson, Gottlieb, Handelsman, Knapp, & Younggren, 2011). Indeed, most recent conceptual advances in the area are explicitly dual process models that integrate the cognitive and emotional aspects of moral decision making and acknowledge that ethical decision making may be driven by emotion and automatic decision-making processes, at least some of the time (Cushman, Young, & Hauser, 2006; Greene, 2007, 2009; Haidt, 2001). The dual processing approach certainly seems more relevant, as is at least sometimes the case that military moral dilemmas need to be resolved rapidly in emerging environments of threat where immediate affective appraisal is likely to dominate rational ethical thinking.

A focus on military ethics seems especially warranted given the media and government reports of the recent (although still relatively rare) incidents of unethical behavior in the militaries of many countries, including Canada, Britain, Australia, the United States, and the Netherlands, to mention just a few (Banks, 2012; De Graff & Van den Berg, 2010; Government of Canada, 1997; Rayment, 2011; Robinson, 2009; Santow, 2011). Concerns in the wake of events such as Abu Ghraib led the US military to commission groundbreaking research (Mental Health Advisory Team (MHAT)-IV, Castro & McGurk, 2007; see also MHAT-V, 2008) that was the first to systematically investigate the battlefield ethical attitudes and behaviors of US soldiers and marines deployed in Iraq and Afghanistan. The unsettling results of that study, though well-known, bear repeating:

Less than half of soldiers and marines believed that noncombatants should be treated with dignity and respect, and well over a third believed that torture should be allowed to save the life of a fellow team member. About 10% of soldiers and marines reported mistreating an Iraqi noncombatant when it wasn't necessary, either by destroying their private property or by hitting or kicking them. Less than half of soldiers or marines would report a team member for unethical behavior, instead preferring to handle it themselves at the team level. (Castro & McGurk, 2007, pp. 29–30)

Although disturbing, these findings were equally revealing in terms of beginning to illuminate a link between unethical attitudes, behaviors, and stress. Specifically, those soldiers who were more likely to have reported unethical attitudes or behaviors were also twice as likely to screen positive for a mental health problem, such as depression, anxiety, or acute stress, or to report higher levels of anger. Level of combat exposure was also associated with unethical attitudes and behaviors. Specifically, soldiers and marines whose units had suffered casualties, or who had handled dead bodies or human remains, were more likely to report that they had verbally abused noncombatants, destroyed civilian property unnecessarily, and in the case of the marine sample, to have reported physically abusing a noncombatant than soldiers and marines whose units did not suffer a casualty or handle human remains (Castro & McGurk, 2007; Warner et al., 2011).

One explanation of these findings is that higher levels of stress associated with deployment events led to ethical violations—an explanation consistent with the emerging laboratory studies of the effects of stressors on moral decision making cited above. Others, however, have suggested that such psychological conflict and suffering is the *result* of an individual perpetrating, witnessing, or failing to prevent actions that contravene his or her fundamental moral beliefs (Cossar, 2008; Litz et al., 2009), and/or societal norms for military personnel (Grossman & Christensen, 2007; Nadelson, 2005). A precondition for such moral injuries (Litz et al., 2009) is the *recognition* of the event as a severe violation of deeply held values that will lead to conflict and stress. However, it is the attribution made concerning the underlying causes of the event that will be necessary for moral injury to ensue. Specifically, if a soldier believes that the causes of the behavior are deemed to be global (as opposed to context specific), internal (i.e., related to a flaw in their character), and stable (i.e., enduring), moral injury, described as alternating between intrusive thoughts and emotions such as guilt or shame and increasingly frantic efforts to avoid same, will ensue. This pattern will play out repeatedly and be associated with long-term conflict and distress. “The more time passes, the more service members will be convinced and confident that not only their actions, but *they* are unforgiveable … [and] will fail to see a path toward renewal and reconciliation; they will fail to forgive themselves and experience self-condemnation” (Litz et al., 2009, p. 698). This evocative description is entirely consistent with the wider literature documenting the importance of the quality of posttrauma narrative on a person's self-evaluation, and the importance of building a constructive and coherent meaning regarding the event in order to process it in a constructive manner (Janoff-Bulman, 1992; Pennebaker & Seagal, 1999; Taylor, 1983; Tuval-Mashiach et al., 2004).

Debates concerning cause and effect relationships notwithstanding—and indeed both explanations are likely to be true at times—results such as these make clear that militaries must remain vigilant about operational ethics. This vigilance is certainly fundamental to militaries’ duty of care to ensure the psychological well-being of individual military members sent into harm's way. However, as noted earlier, such vigilance is also important because the unethical actions of even a few military personnel reduce level of host and home country public support for the mission, undermining operational legitimacy and effectiveness (Stouffer & Seiler, 2010). Finally, given the complexity and myriad stressors inherent in modern operations, there is a strong case to be made that even greater skill in moral judgment, decision making, and action is required to maintain operational effectiveness and soldier well-being in future missions.

Speaking on this issue, a recent volume by leading international military ethicists questioned whether existing military ethics education and training have kept apace of these new ethical complexities (Carrick, Connelly, & Robinson, 2009). Indeed, in the view of these experts, the vast majority of military ethical education has remained rooted in the traditions and value systems of conventional war-fighting, rather than updating curriculums to incorporate these emerging ethical issues. Moreover, despite growing evidence that demonstrates a bidirectional link between stress and ethical behaviors, it continues to be the case that mental health training (Stress and Coping or Resilience) and ethics training are developed and delivered completely, independent of each other. However, it may well be that the mental health training becomes particularly relevant as one confronts ethical decisions and likewise the impact of ethical dilemmas on well-being should be underscored during ethics training.

Such concerns are supported by other empirical findings in Castro and McGurk's research. Although approximately 80% of the soldiers and marines reported receiving ethics training concerning the proper treatment of noncombatants, about 25% of them also reported that they faced ethical situations in Iraq in which they were not sure how to respond. Moreover, Warner et al. (2011) reported that most soldiers were unsatisfied by traditional PowerPoint presentation approaches to operational ethics preparation. Certainly, it seems patently unfair to expect military personnel to respond ethically if they are not provided the most relevant and effective preparation for current missions (Robinson, 2009).

The question then becomes what should ethics preparation for contemporary military missions look like, and what foundational principles should guide its development? Certainly, it would need to develop and exercise the ability to recognize a moral issue (i.e., moral awareness), and moral decision-making skills. Given the reality of military operations, it must avoid an overreliance on exclusively cognitive-based models; it must address the role of stress and situational factors on moral decision making and behaviors, and provide practice in order to mitigate these effects. Indeed, opportunities to practice undertaking moral behaviors, perhaps especially in situations that mirror operational stressors, and also situations that might be constructed to involve some operational challenges is likely critical. Importantly, it would need to accomplish these objectives in ways that will be meaningful and immediately relevant to a majority of military personnel who undertake the training.

Several recent sources reinforce these principles and provide additional insights on how to achieve these objectives. For instance, Johnson (2011) outlined several relevant training objectives for contemporary missions:

1. *Increased moral awareness*: Service members will be able to recognize the moral aspects of an operational setting; service members will be able to understand the relevant moral dimensions in an operational setting; service members will be able to see the moral implications of the decision;
2. *Exercise moral judgment*: Service members will be able to identify appropriate levels of moral responsibility in situations of moral ambiguity or complexity; service members will be able to demonstrate the recognition of different—sometimes competing—cultural moral systems, as active, though not necessarily binding, within different areas of responsibility; service members will be able to identify an appropriate understanding of their role in the encounter and their range of potential responses.
3. *Increased confidence and mastery*: Service members will develop their confidence in confronting morally complex situations;

She further proposed “moral resiliency training,” a highly interactive, carefully constructed and guided approach consisting of decision-making games, hot washes, historical and personal case studies to take place within resident officer professional military education (PME). Importantly, Johnson (2011) advocated that scenarios should be morally ambiguous or complex so that service members are able “to confront the absence of ‘right’ answers, … [and understand that] they may not [always] be able to resolve the dilemma, solve the problem, or ‘do the right thing’” (p. 279), as there are times when this may be the case in operations (Thompson, Adams, & Thomson, 2008). Personnel must have the opportunity to acknowledge that this may be the case and to work out a range of possible responses to such circumstances (Robinson, 2007). Moreover, given the diverse cultures increasingly encountered in contemporary missions, “training must cultivate mechanisms within service members to live in environments with different, even competing moral systems … [including] specific strategies for managing the moral disconnect that members are likely to face during their deployments” (Johnson, 2011, p. 277).

She also argued that PME provides the time for critical thinking skills to analyze moral challenges, using three key psychological processes: situational reconstruction, in which individuals revisit the experience in order to gain perspective; focusing, in which individuals explore their physical reactions to the event; and compensatory self-improvement, in which individuals envision what actions they can now take to develop confidence in their ability to take future action. Also, the peer insights and support, mentor supervision, and access to mental health professionals within resident PME, would allow officers to “create an environment in which officers can process past morally traumatic events, prepare themselves for the morally traumatic situations they may experience during future deployments, and learn how to prepare their subordinates to do the same” (p. 278).

Note that Johnson's suggestion that PME provides the opportunity to process past traumatic events also implies the importance of mental health in this area. Indeed, the issue of the impact of prior traumatic exposures bears some special attention. For instance, it suggests that the “psychic noise” of unprocessed trauma/or symptoms may well have an impact on ethical decisions. Despite Johnson's optimistic assessment of the value of revisiting events in PME, it is also the case that if prior events remain psychically scarring that processing of past traumatic events may be more difficult. This can be further complicated by a coping strategy of cognitive avoidance in order to suppress the earlier memory. There is no doubt that these will have a significant effect on a person's moral decision-making ability. Such a pattern could highlight particular at-risk individuals or groups who may be more vulnerable in this regard. In particular, it may suggest the necessity for engaged leadership (Warner & Appenzeller, 2011) who are attuned to the attitudes and behaviors of their soldiers after traumatic events such as the loss of a comrade.^1^

Second, in light of the serious issues illuminated by the MHAT-IV and MHAT-V studies, the US military developed battlefield ethics training to be administered during a deployment (Warner et al., 2011). The training, occurring 7–8 months into the year-long deployment, involved viewing selected movie clips that depicted military moral dilemmas involving violations similar to those highlighted by the MHAT findings (e.g., treatment of noncombatants, and reporting of ethical failures). This was followed by semi-standardized (i.e., key discussion points and critical questions were provided) leader-guided small group discussions of the ethical issues raised and the ways in which these issues might be addressed. A large-scale program evaluation spoke to the program's apparent success. Results showed statistically significant decreases in reports of unethical attitudes and behaviors related to the treatment of noncombatants and civilian property, significant decreases in the reports of witnessing unethical behaviors by other soldiers, and significant increases in soldiers’ willingness to report the ethical violations of peers, as compared to these same soldiers’ pretraining MHAT responses. Finally, analyses revealed the major predictor of the majority of unethical attitudes and behaviors to be amount of combat exposure rather than mental distress per se, although PTSD and time outside the wire continued to be somewhat related to cursing and yelling at noncombatants, even after the effects of combat exposure were accounted for.

These are approaches undoubtedly useful in addressing two points in the continuum of moral preparation for military operations: PME and during a deployment. However, several noted military ethicists also make a compelling argument for the importance of an operational ethics training component as part of soldiers’ high-intensity preparation for operations: “[E]thics education should not be seen as something for the classroom only” (Robinson, 2009, p. 9). Lucas went even further, noting that “ungrounded and untested raw intuitions can differ substantially, and provide little in the way of guidance to individuals facing stark choices in the heat of conflict” (Lucas, 2009, p. xii). Similar thinking also led McMaster (2009) to conclude that effective ethical preparation must include “tough, realistic training appropriate for the environment that soldiers will face” (p. 15), a concern particularly relevant given the myriad stressors, ambiguity, and intensity of complex modern military operations.

When considering the issue of integrating moral decision-making training into high-intensity training environments, several lessons from Thompson and McCreary's (2006) mental readiness training are also relevant. Their approach was developed specifically in response to some of the limitations of traditional military stress management training, including the reliance on lecture formats and PowerPoint briefings delivered in settings distinct from operational training, and presented by mental health professionals with little operational experience. The result of this traditional stress management approach was that important information was often seen as minimally relevant to the ongoing experience of deployed forces, presented in a format that was not engaging, and presented by instructors who lacked operational credibility; all of which ultimately hampered the effectiveness of these programs (Thompson & Pastò, 2003). In contrast, the mental readiness approach is based on a more seamless integration of skills application into selected, high intensity, military training environments. The idea was to embed lessons and training points in operationally relevant contexts so that skill acquisition and rehearsal are more intrinsically applicable and salient to soldiers, the skills more contiguously practiced, and the results more immediately experienced. Based in the cognitive behavioral tradition, the training approach seeks to emphasize the interconnection between cognitive, physiological, and emotional systems; acknowledging that arousal in one system can lead to increased arousal in the remaining systems through a series of feedback loops (Mischel, 2004). This principle clearly echoes the military findings best illustrated by the MHAT data that indicate a link between stress and decision making, and incorporating the potential for bidirectional effects (high stress leading to moral decision-making errors, i.e., Castro & McGurk, 2007, and/or moral decision-making errors leading to higher and longer-term stress outcomes, i.e., Litz et al., 2009). And indeed most recently, concerted efforts by Canadian Forces’ mental health professionals have transformed traditional mental health training into Resilience Training, using the “Big 4” from sports psychology—goal setting, mental rehearsal/visualization/self-talk and arousal reduction/tactical breathing (Crust & Azadi, 2010). Just as these skills quiet the noise of stress when an athlete faces challenges, it is designed in part, to assist soldiers when facing the enemy (Department of National Defense, 2013). We also believe that such skills may also play a role in quieting the surrounding psychic noise in order to make an ethical decision, although this is a hypothesis that needs to be confirmed with empirical research.

Integrating these various sources then, such scenario-based moral decision-making training would involve carefully constructed scenarios designed to exercise specific moral decision-making challenges that would occur in selected high-intensity training environments. Finally, predeployment training is an ideal place to integrate compelling moral decision-making challenges, wherever possible garnered from the operational experience of veterans of relevant recent missions, into the overall training objectives of such confirmation exercises. This would allow for moral decision-making processes to be used under close to real-life stress conditions, and also provide *in-situ* experience in considering moral implications and options, and balancing these in the context of multiple, potentially competing, operational objectives. Integration of moral decision-making feedback within after-action reviews, feedback from exercise mentors, and discussions among unit members all would be critical to deepening an understanding of moral decision making during the intensity of operations in general; demonstrating how stressors may affect that decision making, and considering the issues and responses relevant to the specific moral scenarios selected for inclusion in training. The careful selection of moral challenges, either drawn directly from or tied to recent operational experiences, is common to both to principles underlying the approaches used by Johnson and by Warner et al.

Although beyond the scope of this paper to discuss in detail, clearly the influences of peers and leaders are critical to the success of this approach. Seminal social psychological theory and research has made clear how (often-related) phenomenon such as obedience and the role of authority (Milgram, 1963; Blass, 1991), peer pressure (Asch, 1951), groupthink (Janis, 1971), social comparison (Festinger, 1954), social learning (Bandura, 1962), social influence (Kelman, 1958), and diffusion of responsibility and bystander effects (Darley & Latane, 1968) can profoundly affect a variety of attitudes and behavior, findings echoed by military ethicist Cossar (2008). “Even though the individual soldier may believe in ethical treatment of others, it is easy to get pulled into the killing. Fear, authority, dehumanization, and indoctrination are just some of the factors that may lead people to react against their will” (p. 31). In addition, interactions with peers typically involve a large emotional component which, magnified by the heat of battle, further triggers automatic processing. For various reasons then, important others may often outweigh the influence of moral principles in such situations (Betan & Stanton, 1999) and affect evaluations of the unethical behavior of significant others (Gino, Moore, & Bazerman, 2008) especially in cases where the resultant harm is not clear, immediate, and direct; and when there has been a slow erosion of a culture of ethics within a group; all heuristic biases that could be further heightened under stressful conditions. A related but crucial issue in this regard is the dilemma for the individual who raises a moral or ethical criticism of his colleagues in a combat environment. Given that these may lead to legal investigations, it is important that such scenarios are equally addressed. The potential alienation and bullying of individuals who raise complex moral issues in the combat environment need to be an integrated part of any effective program.

Although predeployment training is obviously too late for a first introduction to these issues, the scenario-based training experience of integrating at least some of these social influences at play in terms of operational ethics may be one way to further mitigate their potential negative impacts while fostering their positive effects. Consistent with the empirical findings of Warner and colleagues, those principles that cannot be directly integrated into the moral scenario must be dealt with explicitly in post-scenario unit discussions, led by unit leaders and/or trainers with the requisite operational experience.

In the final analyses, the current approach is predicated on the actual rehearsal of important moral decision processes, in contexts as close as possible to operational environments, with all of their attendant stresses in accordance with Robinson (2009), Lucas (2009), and McMaster (2009). As with any skill, rehearsal is fundamental to building a better understanding of relevant issues and responses for the best course of action. This further builds a sense of certainty, control, and confidence (Cossar, 2008; Johnson, 2011), while reducing interference from competing responses (Driskell et al., 2006), which may be particularly important in complex tasks (Keinan & Friedland, 1996) such as those inherent in recent missions. This enables valuable learning in realistic environments, but also allows for the possibility of learning in the absence of true harm to self, others, or the mission. For all of these reasons then we believe that integrating moral situations within high-intensity military training settings should contribute to the development of the “mental and emotional conditioning needed to respond [appropriately to moral issues] in combat” (Johnson, 2011, p. 279).

## Concluding thoughts

We have presented evidence from a variety of sources to make the case that the integration of ethical scenarios within high-intensity field training will benefit soldier psychological well-being and operational effectiveness. However, we are quick to point out that this approach should not be treated as a panacea. Indeed, one explicit objective of the approach that is outlined here (and of all moral education and training) is increased moral awareness. Ignorance can be bliss: thus a potential risk of this, and indeed all engaging moral training and education, is the real possibility that some soldiers may be more vulnerable as a result of this training, at least initially. It bears repeating therefore, that engaged leaders and peers are invaluable to keeping watch for signs of confusion or distress in peers, similar to that undertaken with respect to many technical military skills. In the end, we believe that the benefits accrued by the careful development of moral decision-making skills in response to relevant operational scenarios, practiced with the leaders and peers with whom one will deploy, in realistic, but nonetheless safe conditions (where “do overs” are possible), is anticipated to mitigate many of these risks, while better addressing the significant and long-term liabilities that remain if such approaches are not adopted.

We are not advocating that conventional moral education be abandoned. On the contrary, PME lays an important, thoughtful grounding in the values and factors that must be taken into consideration in military ethics (Bradley, 2010), and may be particularly important in settings where rational and deliberative decision-making processes are possible. What we are suggesting here is that these educationally-based approaches alone are not enough. Similarly, military ethicists continue to have a crucial place in education and as part of the operational ethics training team. However, within high-intensity scenario-based training, we believe that the important issues associated with operational ethics may resonate more if delivered by military personnel with acknowledged operational experience and credibility. Given the importance of moral behaviors to soldier well-being and mission effectiveness, the ideal approach would involve a deliberate, integrated program of moral preparation for operations including PME activities discussed by Johnson (2011), focused high-intensity predeployment training outlined here, and in-theater training as developed by Warner et al. (2011).

We recognize that some trainers and unit leaders will have addressed the issue of operational ethics wherever they can; for instance, encouraging trainees to think about the moral aspects of particular situations. What we propose here is an expansion of often *ad hoc* opportunities to a more systematic and seamless integration of moral decision-making training within relevant high-intensity military predeployment training scenarios. In this way, operational ethics training objectives more explicit and demonstrate organizational commitment to this objective—indeed training of this nature will most certainly fail if training staff, peers, and particularly unit leaders and the organization does not seem to endorse it.

There is also no doubt that what we are outlining here adds burdens to military training systems and schedules that are already overstretched in providing currently mandated training. The approach requires that course designers and instructors pay increasing attention to effectively incorporating moral decision making into selected training, in addition to the wide range of other objectives they are already expected to cover. Furthermore, as the most powerful training would take place with scenarios taken, or combining elements from theater after-action reports, it will also require that lessons learned collection must be expanded to include gathering of relevant moral and ethical decision-making challenges in operations, and greater communication between lessons learned and training design personnel. Finally, we acknowledge that we are suggesting these additions against the current backdrop of sizable cuts to defense budgets, with their increased pressure on military organizations to reduce training time and costs. The hope is that the focus on the integration of this approach into selected ongoing training activities will reduce at least some of the associated time and financial costs in the long run.

In fact, some militaries have recently integrated moral decision-making challenges into selected high-intensity training in a manner consistent with what we have outlined here. For instance, the Canadian Forces adopted such an approach in the wake of the difficult ethical challenges arising within the international mission in Afghanistan. The Canadian Manoeuvre Training Centre (CMTC) injected a series of ethical scenarios into Exercise Maple Guardian, a 3-week, complex, large-scale, fully immersive, live-action field training event that was designed to replicate the conditions as much as possible and was the culminating collective training event for the Canadian Battle Group prior to deployment to Afghanistan.

The ethical injects took place while troops were immersed in this high-intensity training environment, patrolling, assisting with reconstruction, and/or mentoring members of the Afghan National Police or Army. Members at any level could encounter a woman being beaten, or hear of sexual abuse, hazing, and/or theft by some of the Afghani security forces. Exercise controllers determine how effectively the inject was handled by all members of the primary training audience (PTA): Does the incident get reported or responded to properly at every level? If there are shortfalls in the reaction, the exercise controllers can either repeat the event, insert another ethical inject, or stop the action to talk to the leadership concerning the training objective and appropriate reactions. This is then explained to the soldiers directly involved in the event and is part of the more formal debriefing of the PTA. Colonel Bernd Horn, then Chief of Staff of the Land Force Doctrine and Training System, summed up the intent of this training:

This ensures that we put a practical test to all the theory education and training. We ensure that they understand the meaning of the words and that they have an obligation to do something. It also provides a vehicle whereby we can reinforce the proper behavior and we can also correct behavior that we think might not be as efficient or effective as we would have liked.

Testimony at the Proceedings of the Canadian Standing Senate Committee on Human Rights, October 19, 2009.

In conclusion, although intense, realistic training is a part of every military culture, what appears to be lacking, and what we are advocating here, is the careful construction and effective integration of, and practice in, key aspects of moral decision making within these high-intensity operational training settings. This will ensure that lessons and training points, skill acquisition, and rehearsal are more intrinsically applicable and salient to soldiers, the skills more contiguously practiced, and the results more immediately experienced in operationally relevant contexts. It is also intended to better ensure that moral dimensions will be activated and considered, and that moral behaviors will be well-rehearsed by the time soldiers are confronted with the myriad intense stressors and ethical challenges that are part of modern, high intensity, complex missions.

## Footnotes

1 The authors thank an anonymous reviewer for raising this issue.
